# Handwritten Bangla Character Recognition Using the State-of-the-Art Deep Convolutional Neural Networks

**DOI:** 10.1155/2018/6747098

**Published:** 2018-08-27

**Authors:** Md Zahangir Alom, Paheding Sidike, Mahmudul Hasan, Tarek M. Taha, Vijayan K. Asari

**Affiliations:** ^1^Department of Electrical and Computer Engineering, University of Dayton, Dayton, OH, USA; ^2^Department of Earth and Atmospheric Sciences, Saint Louis University, St. Louis, MO, USA; ^3^Comcast Labs, Washington, DC, USA

## Abstract

In spite of advances in object recognition technology, handwritten Bangla character recognition (HBCR) remains largely unsolved due to the presence of many ambiguous handwritten characters and excessively cursive Bangla handwritings. Even many advanced existing methods do not lead to satisfactory performance in practice that related to HBCR. In this paper, a set of the state-of-the-art deep convolutional neural networks (DCNNs) is discussed and their performance on the application of HBCR is systematically evaluated. The main advantage of DCNN approaches is that they can extract discriminative features from raw data and represent them with a high degree of invariance to object distortions. The experimental results show the superior performance of DCNN models compared with the other popular object recognition approaches, which implies DCNN can be a good candidate for building an automatic HBCR system for practical applications.

## 1. Introduction

Automatic handwriting character recognition has many academic and commercial interests. The main challenge in handwritten character recognition is to deal with the enormous variety of handwriting styles by different writers. Furthermore, some complex handwriting scripts comprise different styles for writing words. Depending on the language, characters are written isolated from each other in some cases (e.g., Thai, Laos, and Japanese). In some other cases, they are cursive and sometimes characters are related to each other (e.g., English, Bangladeshi, and Arabic). This challenge has been already recognized by many researchers in the field of natural language processing (NLP) [1–3].

Handwritten character recognition is more challenging compared with the printed forms of character due to the following reasons: (1) Handwritten characters written by different writers are not only nonidentical but also vary in different aspects such as size and shape; (2) numerous variations in writing styles of individual character make the recognition task difficult; (3) the similarities of different character in shapes, the overlaps, and the interconnections of the neighbouring characters further complicate the character recognition problem. In summary, a large variety of writing styles and the complex features of the handwritten characters make it a challenge to accurately classifying handwritten characters.

Bangla is one of the most spoken languages and ranked fifth in the world and spoken by more than 200 million people [4, 5]. It is the national and official language of Bangladesh and the second most popular language in India. In addition, Bangla has a rich heritage. February 21st is announced as the International Mother Language day by UNESCO to respect the language martyrs for the language in Bangladesh in the year of 1952. In terms of Bangla character, it involves a Sanskrit-based script that is inherently different from English- or Latin-based scripts, and it is relatively difficult to achieve desired accuracy on the recognition tasks. Therefore, developing a recognition system for Bangla characters is of a great interest [4, 6, 7].

In Bangla language, there are 10 digits and 50 characters including vowel and consonant, where some contain additional sign up and/or below. Moreover, Bangla consists of many similar shaped characters. In some cases, a character differs from its similar one with a single dot or mark. Furthermore, Bangla also contains some special characters with equivalent representation of vowels. This makes it difficult to achieve a better performance with simple classification technique as well as hinders to the development of a reliable handwritten Bangla character recognition (HBCR) system.

**Figure 1:**
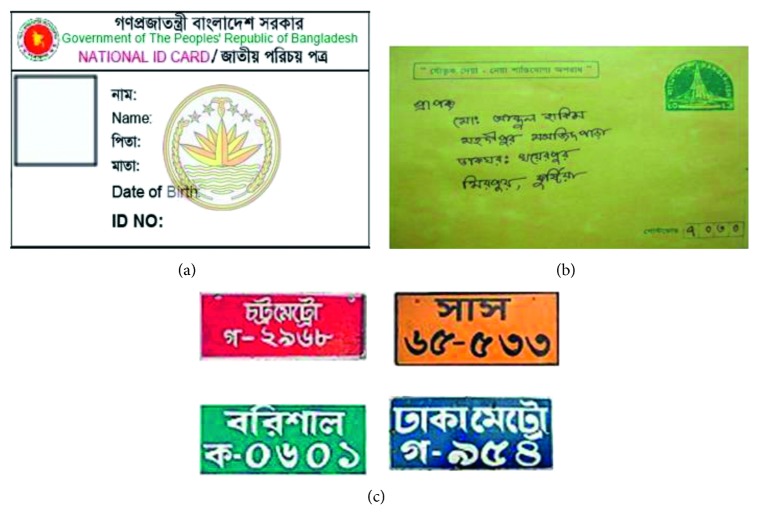
Application of handwritten character recognition: (a) national ID number recognition system, (b) post office automation with code number recognition on envelope, and (c) automatic license plate recognition.

There are many applications of HBCR, such as Bangla optical character recognition, national ID number recognition system, automatic license plate recognition system for vehicle and parking lot management system, post office automation, and online banking. Some example images of these applications are shown in Figure 1. In this work, we investigate the HBCR on Bangla numerals, alphabets, and special characters using the state-of-the-art deep convolutional neural network (DCNN) [8] models. The contributions of this paper can be summarized as follows:

- First time to comprehensive evaluation of the state-of-the-art DCNN models including VGG Net [9], All Convolutional Neural Network (All-Conv) [10], Network in Network (NiN) [11], Residual Network (ResNet) [12], Fractal Network (FractalNet) [13], and Densely connected convolutional Network (DenseNet) [14] on the application of HBCR.
- Extensive experiments on HBCR including handwritten digits, alphabets, and special character recognition.
- The better recognition accuracy is achieved, to the best of knowledge, compared with other existing approaches that reported in the literature.

## 2. Related Work

Although some studies on Bangla character recognition have been reported in the past years [15–17], there is a few remarkable works available for HBCR. Pal and Chaudhuri [5] proposed a new feature extraction-based method for handwritten Bangla character recognition where the concept of water overflow from the reservoir is utilized. Liu and Suen [18] introduced directional gradient features for handwritten Bangla digit classification using ISI Bangla numeral dataset [19], which consists of 19,392 training samples, 4000 test samples, and 10 classes (i.e., 0 to 9). Surinta et al. [20] proposed a system using a set of features such as the contour of the handwritten image computed using 8-directional codes, distance calculated between hotspots and black pixels, and the intensity of pixel space of small blocks. Each of these features is separately fed into support vector machine (SVM) [21] classifier, and the final decision is made by the majority voting strategy. Das et al. [22] exploited genetic algorithms-based region sampling method for local feature selection and achieved 97% accuracy on HBCR. Xu et al. [23] used a hierarchical Bayesian network which directly takes raw images as the network inputs and classifies them using a bottom-up approach. Sparse representation classifier has also been applied for Bangla digit recognition [4], where 94% accuracy was reported for handwritten digit recognition. In [6], handwritten Bangla basic and compound character recognition using multilayer perceptron (MLP) [24] and SVM classifier was suggested, while handwritten Bangla numeral recognition using MLP was presented in [7] where the average recognition rate reached 96.67%.

Recently, deep learning-based methods have drawn increasing attention in handwritten character recognition [25, 26]. Ciregan and Meier [27] applied multicolumn CNNs to Chinese character classification. Kim and Xie [25] applied DCNN to Hangul handwritten character recognition and superior performance has been achieved against classical methods. A deep learning framework such as a CNN-based HBCR scheme was introduced in [26] where the best recognition accuracy reached at 85.36% on their own dataset. In this paper, we, for the first time, introduce the very latest DCNN models, including VGG network, All-Conv, NiN, ResNet, FractalNet, and DenseNet, for handwritten Bangla character (e.g., digits, alphabets, and special characters) recognition.

## 3. Deep Neural Networks

Deep neural network (DNN) is an active area in the field of machine learning and computer vision [28] and it generally contains three popular architectures: Deep Belief Net (DBN) [29], Stacked Autoencoder (SAE) [30], and CNN. Due to the composition of many layers, DNN methods are more capable for representing the highly varying nonlinear function compared with shallow learning approaches [31]. The low and middle level of DNN abstract the feature from the input image, whereas the high level performs classification operation on the extracted features. As a result, an end-to-end framework is formed by integrating with all necessary layers within a single network. Therefore, DNN models often lead to better accuracy compared with the other type of machine learning methods. Recent successful practice of DNN covers a variety of topics such as electricity consumption monitoring [32], radar signal examination [33], medical image analysis [34–36], food security [37–39], and remote sensing [40–42].

Among all deep learning approaches, CNN is one of the most popular models and has been providing the state-of-the-art performance on segmentation [43, 44], human action recognition [45], image superresolution [46], scene labelling [47], and visual tracking [48].

### 3.1. Convolutional Neural Network (CNN)

CNN was initially applied to digit recognition task by LeCun et al. [8]. CNN and its variants are gradually adopted to various applications [46, 49]. CNN is designed to imitate human visual processing, and it has highly optimized structures to process 2D images. Furthermore, CNN can effectively learn the extraction and abstraction of 2D features. In detail, the max-pooling layer of CNN is very effective in absorbing shape variations. Moreover, sparse connection with tied weights makes CNN involve with fewer parameters than a fully connected network with similar size. Most importantly, CNN is trainable with the gradient-based learning algorithm and suffers less from the diminishing gradient problem. Given that the gradient-based algorithm trains the whole network to minimize an error criterion directly, CNN can produce highly optimized weights and good generalization performance [50].

**Figure 2:**
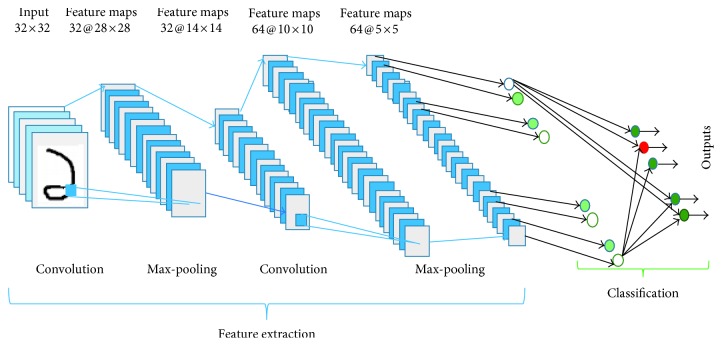
Basic CNN architecture for digit recognition.

The overall architecture of a CNN, as shown in Figure 2, consists of two main parts: feature extractor and classifier. In the feature extraction unit, each layer of the network receives the output from its immediate previous layer as inputs and passes current output as inputs to the immediate next layer, whereas classification part generates the predicted outputs associated with the input data. The two basic layers in CNN architecture are convolution and pooling [8] layers. In convolution layer, each node extracts the features from the input images by convolution operation on the input nodes. The max-pooling layer abstracts the feature through average or maximum operation on input nodes. The outputs of *l* − 1th layer are used as input for the *l*th layer, where the inputs go through a set of kernels followed by nonlinear function ReLU. Here, *f* refers to activation function of ReLU. For example, if *x*_*i*_^*l*−1^ inputs from *l* − 1th layer, *k*_*i*,*j*_^*l*^ are kernels of *l*th layer. The biases of *l*th layer are represented with *b*_*j*_^*l*^. Then, the convolution operation can be expressed as

(1) $$\begin{array}{c} x_{j}^{l}=f\left(x_{i}^{l-1}*k_{i,j}^{l}\right)+b_{j}^{l}. \end{array}$$

The subsampling or pooling layer abstracts the feature through average or maximum operation on input nodes. For example, if a 2 × 2 down sampling kernel is applied, then each output dimension will be the half of the corresponding input dimension for all the inputs. The pooling operation can be stated as follows:

(2) $$\begin{array}{c} x_{j}^{l}=\text{down}\left(x_{i}^{l-1}\right). \end{array}$$

In contrast to traditional neural networks, CNN extracts low- to high-level features. The higher-level features can be derived from the propagated feature of the lower-level layers. As the features propagate to the highest layer, the dimension of the feature is reduced depending on the size of the convolution and pooling masks. However, the number of feature mapping usually increased for selecting or mapping the extreme suitable features of the input images for better classification accuracy. The outputs of the last layer of CNN are used as inputs to the fully connected network and it typically uses a Softmax operation to produce the classification outputs. For an input sample **x**, weight vector **w**, and *K* distinct linear functions, the Softmax operation can be defined for the *i*th class as follows:

(3) $$\begin{array}{c} P\left(\left.y=i\right|x\right)=\frac{\exp\left(x^{T}w_{i}\right)}{\sum_{k=1}^{K}\exp\left(x^{T}w_{k}\right)}. \end{array}$$

However, there are different variants of DCNN architecture that have been proposed over the last few years. The following section discusses six popular DCNN models.

### 3.2. CNN Variants

As far as CNN architecture is concerned, it can be observed that there are some important and fundamental components that are used to construct an efficient DCNN architecture. These components are convolution layer, pooling layer, fully connected layer, and Softmax layer. The advanced architecture of this network consists of a stack of convolutional layers and max-pooling layers followed by fully connected and Softmax layer at the end. Noticeable examples of such networks include LeNet [8], AlexNet [49], VGG Net, All-Conv, and NiN. There are some other alternative and advanced architecture that have been proposed, including GoogleNet with inception layers [51], ResNet, FractalNet, and DenseNet. However, there are some topological differences observed in the modern architectures. Out of many DCNN architectures, AlexNet, VGG Net, GoogleNet, ResNet, DenseNet, and FractalNet can be viewed as most popular architectures with respect to their enormous performance on different benchmarks for object classification. Among these models, some of the models are designed especially for large-scale implementation such as ResNet and GoogleNet, whereas the VGG Net consists of a general architecture. On the other hand, FractalNet is an alternative of ResNet. In contrast, DenseNet's architecture is unique in terms of unit connectivity where every layer to all subsequent layers is directly connected. In this paper, we provide a review and comparative study of All-Conv, NiN, VGG-16, ResNet, FractalNet, and DenseNet for Bangla character recognition. The basic overview of these architectures is given in the following section.

#### 3.2.1. VGG-16

**Figure 3:**
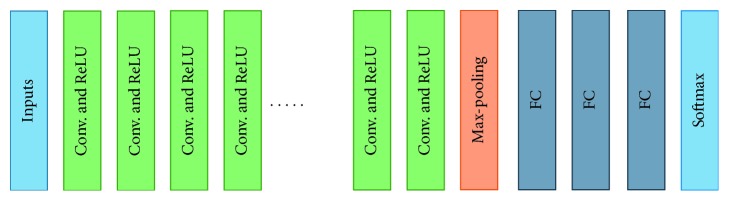
Basic architecture of VGG Net: convolution (Conv) and FC for fully connected layers and Softmax layer at the end.

The visual geometry group (VGG) was the runner up of the ImageNet Large Scale Visual Recognition Competition (ILSVRC) in 2014 [52]. In this architecture, two convolutional layers are used consecutively with a rectified linear unit (ReLU) [53] activation function followed by single max-pooling layer, several fully connected layers with ReLU and Softmax as the final layer. There are three types of VGG Net based on the architecture. These three networks contain 11, 16, and 19 layers and named as VGG-11, VGG-16, and VGG-19, respectively. The basic structure for VGG-11 architecture contains eight convolution layers, one max-pooling layer, and three fully connected (FC) layers followed by single Softmax layer. The configuration of VGG-16 is as follows: the number of convolutions and max-pooling layers: 13, max-pooling layer: 1, FC layers: 3, and Softmax layer: 1. Total weights is 138 million. The VGG-19 consisted of 16 convolutional layers, one max-pooling layer, 3 FC layers followed by a Softmax layer. The basic building blocks of VGG architecture is shown in Figure 3. In this implementation, we have used VGG-16 network with less number of feature maps in convolutional layers compared with the standard VGG-16 network.

#### 3.2.2. All Convolutional Network (All-Conv)

**Figure 4:**
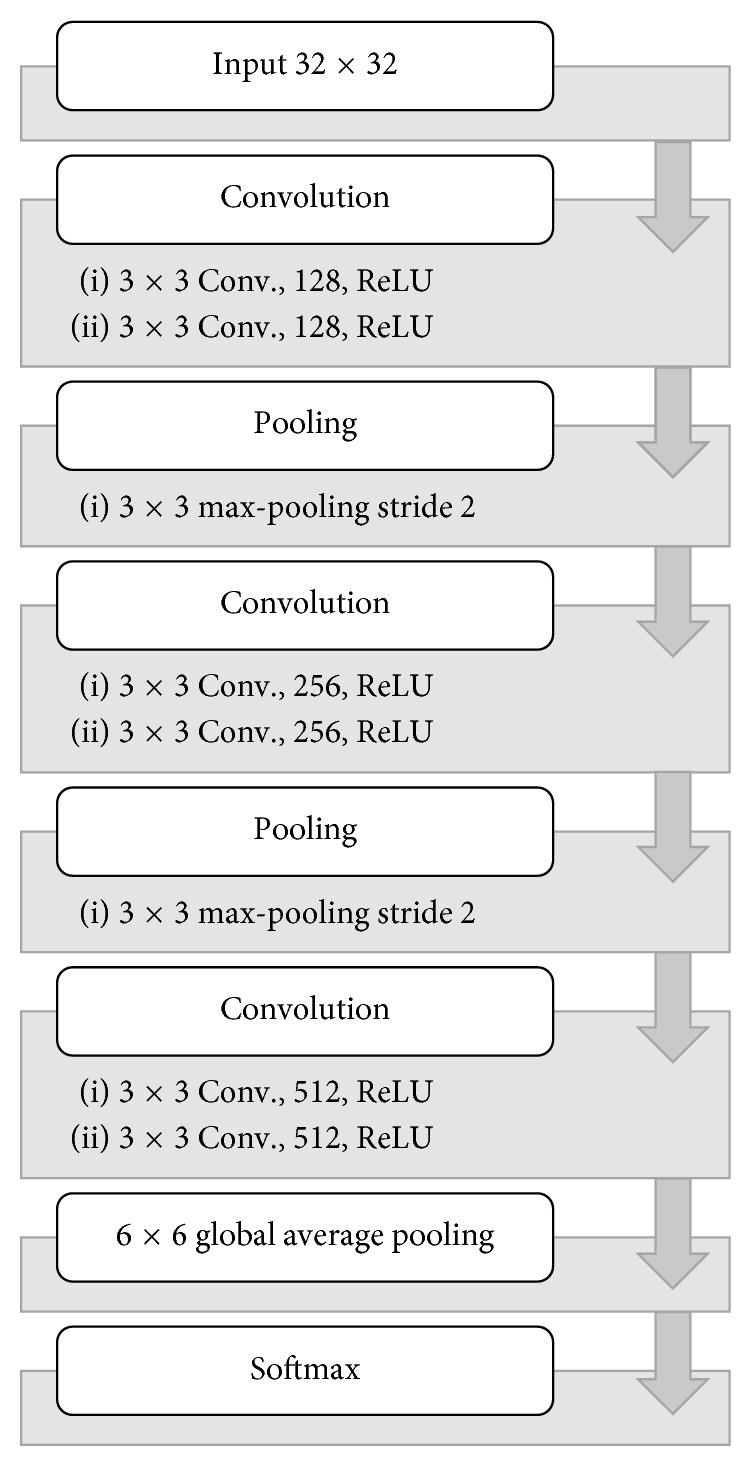
All convolutional network framework.

The layer specification of All-Conv is given in Figure 4. The basic architecture is composed with two convolutional layers followed by a max-pooling layer. Instead of using fully connected layer, global average pooling (GAP) [11] with the dimension of 6 × 6 is used. Finally, the Softmax layer is used for classification. The output dimension is assigned based on the number of classes.

#### 3.2.3. Network in Network (NiN)

This model is quite different compared with the aforementioned DCNN models due to the following properties [11]:

- It uses multilayer convolution where convolution is performed with 1 × 1 filters.
- It uses GAP instead of fully connected layer.

The concept of using 1 × 1 convolution helps to increase the depth of the network. The GAP significantly changes the network structure, which is used nowadays very often as a replacement of fully connected layers. The GAP on a large feature map is used to generate a final low-dimensional feature vector instead of reducing the feature map to a small size and then flattening the feature vector.

#### 3.2.4. Residual Network (ResNet)

**Figure 5:**
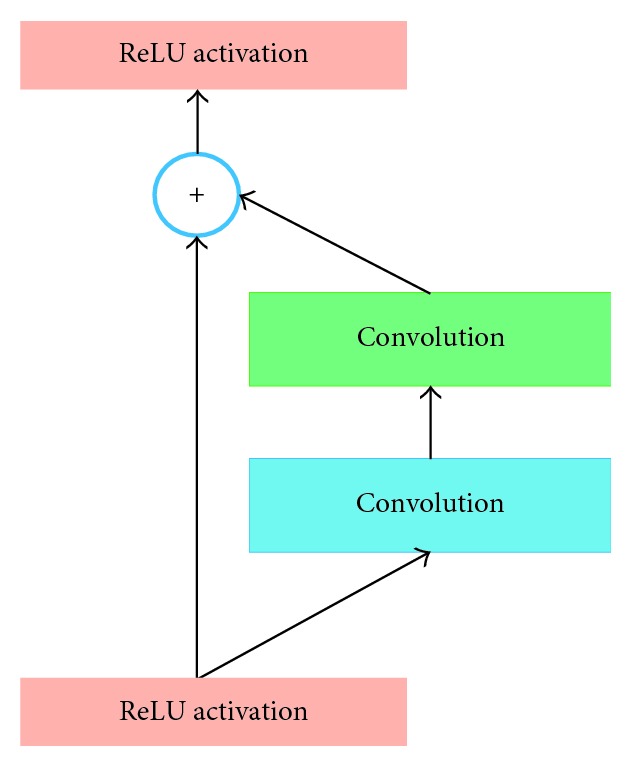
Basic diagram of residual block.

ResNet architecture becomes very popular in computer vision community. The ResNet variants have been experimented with different number of layers as follows: number of convolution layers: 49 (34, 152, and 1202 layers for other versions of ResNet), number of fully connected layers: 1, weights: 25.5 M. The basic block diagram of ResNet architecture is shown in Figure 5. If the input of the residual block is *x*_*l*−1_, the output of this block is *x*_*l*_. After performing operations (e.g., convolution with different size of filters, batch normalization (BN) [54] followed by a activation function such as ReLU) on *x*_*l*−1_, the output *F*(*x*_*l*−1_) is produced. The final output of the residual unit is defined as

(4) $$\begin{array}{c} x_{l}=F\left(x_{l-1}\right)+x_{l-1}. \end{array}$$

The Residual Network consists of several basic residual units. The different residual units are proposed with different types of layers. However, the operations between the residual units vary depending on the architectures that are explained in [12].

#### 3.2.5. FractalNet

**Figure 6:**
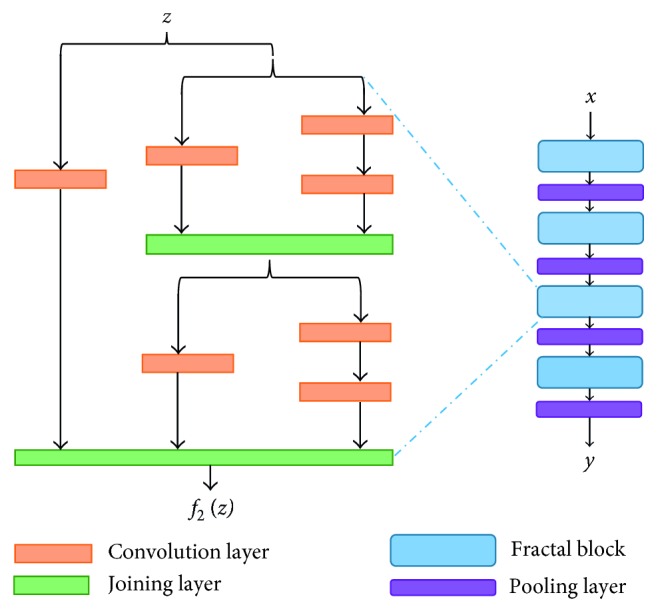
An example of FractalNet architecture.

The FractalNet architecture is an advanced and alternative one of ResNet, which is very efficient for designing very large network with shallow subnetworks, but shorter paths for the propagation of gradient during training [13]. This concept is based on drop path which is another regularization for large network. As a result, this concept helps to enforce speed versus accuracy tradeoff. The basic block diagram of FractalNet is shown in Figure 6. Here *x* is the actual inputs of FractalNet, and *z* and *f*(*z*) are the inputs and outputs of Fractal block, respectively.

#### 3.2.6. Densely Connected Network (DenseNet)

**Figure 7:**
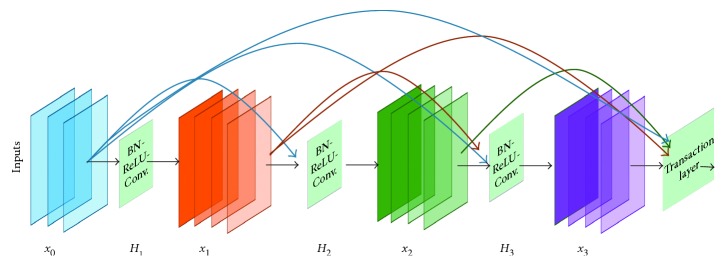
A 4-layer dense block with growth rate of *k* = 3. Each of the layers takes all of the preceding feature maps as input.

DenseNet is densely connected CNN where each layer is connected to all previous layers [14]. Therefore, it forms very dense connectivity between the layers and so it is called DenseNet. The DenseNet consists of several dense blocks, and the layer between two adjacent blocks is called transition layers. The conceptual diagram of the dense block is shown in Figure 7. According to the figure, the *l*th layer receives all the feature maps *x*_0_, *x*_1_, *x*_2_, …, *x*_*l*−1_ from the previous layers as input, which is expressed by

(5) $$\begin{array}{c} x_{l}=H_{l}\left(\left[x_{0},\;\;x_{1},\;\;x_{2},\;\;\ldots ,\;\;x_{l-1}\right]\right), \end{array}$$

where [*x*_0_, *x*_1_, *x*_2_, …, *x*_*l*−1_] is the concatenated features from 0, …, *l* − 1 layers and *H*_*l*_(·) is a single tensor. DenseNet performs three consecutive operations, BN, followed by ReLU and a 3 × 3 convolution. In the transition block, 1 × 1 convolutional operations are performed with BN followed by 2 × 2 average pooling layer. This new architecture has achieved state-of-the-art accuracy for object recognition on the five different competitive benchmarks.

#### 3.2.7. Network Parameters

The number of network parameters is a very important criterion to assess the complexity of the architecture. The number of parameters can be used to make comparison between different architectures. At first, the dimension of the output feature map can be computed as

(6) $$\begin{array}{c} M=\frac{N-F}{S}+1, \end{array}$$

where *N* denotes the dimension of input feature maps, *F* refers to the dimension of filters or receptive field, *S* represents stride in the convolution, and *M* is the dimension of output feature maps. The number of parameters (without bias) for a single layer is obtained by

(7) $$\begin{array}{c} P_{l}=\left(F\times F\times FM_{l-1}\right)\times FM_{l}, \end{array}$$

where *P*_*l*_ represents the total number of parameters in the *l*th layer, *FM*_*l*_ is the total number of output feature maps of *l*th layer, and *FM*_*l*−1_ is the total number of feature maps in the (*l* − 1)th layer. For example, let a 32 × 32 dimensional (*N*) image be an input. The size of the filter (*F*) is 5 × 5 and stride (*S*) is 1 for convolutional layer. The output dimension (*M*) of the convolutional layer is 28 × 28 which is calculated according to (6). For better illustration, a summary of parameters used in All-Conv architecture is shown in Table 1. Note that the number of trainable parameters is zero in the pooling layer.

## 4. Results and Discussion

The entire experiment is performed on desktop computer with Intel® Core-I7 CPU @ 3.33 GHz, 56.00 GB memory, and Keras with Theano on the backend on Linux environment. We evaluate the state-of-the-art DCNN models on three datasets from CMATERdb (available at: https://code.google.com/archive/p/cmaterdb/) containing Bangla handwritten digits, alphabets, and special character recognition.

The statistics of three datasets used in this paper are summarized in Table 2. For convenience, we named the datasets as Digit-10, Alphabet-50, and SpecialChar-13, respectively. All images are rescaled to 32 × 32 pixels in our experiment.

### 4.1. Bangla Handwritten Digit Dataset

**Figure 8:**
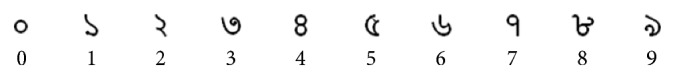
First row shows the Bangla actual digits and second row shows the corresponding Arabic numerals.

**Figure 9:**
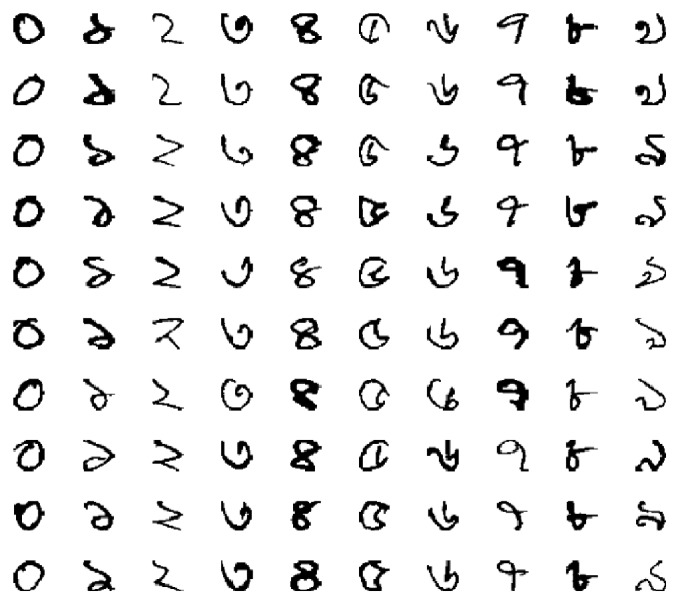
Sample handwritten Bangla numeral images from CMATERdb 3.1.1 database, including digits from 1 to 10.

The standard samples of the numeral with respective Arabic numerals are shown in Figure 8. The performance of both DBN and CNN is evaluated on a Bangla handwritten benchmark dataset called CMATERdb 3.1.1 [22]. This dataset contains 6,000 images of unconstrained handwritten isolated Bangla numerals. Each digit has 600 images that are rescaled to 32 × 32 pixels. Some sample images in the database are shown in Figure 9. Visual inspection depicts that there is no visible noise. However, variability in writing style is quite high due to user dependency. In our experiments, the dataset is split into a training set and a test set for the evaluation of different DCNN models. The training set consists of 4,000 images (400 randomly selected images of each digit). The rest of the 2,000 images are used for testing.

**Figure 10:**
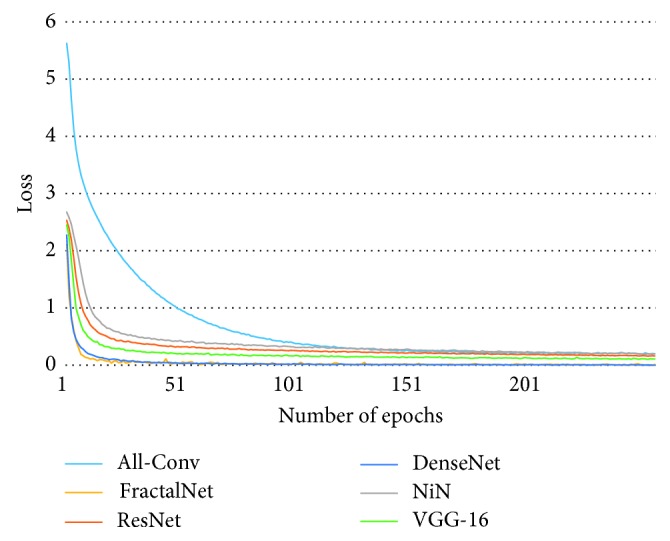
Training loss of different architectures for Bangla handwritten 1–10 digits.

**Figure 11:**
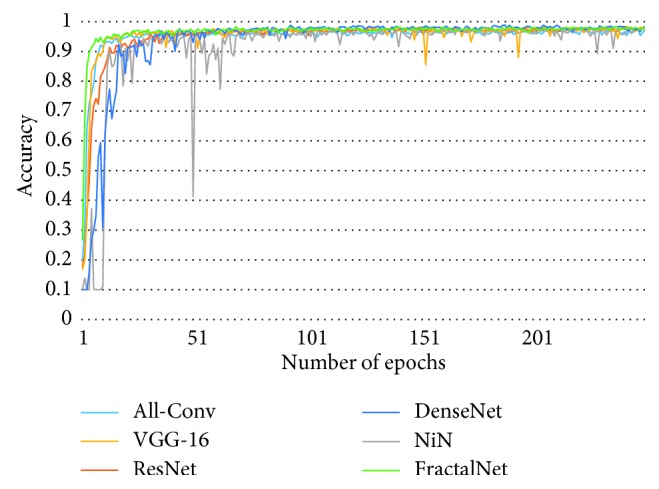
Validation accuracy of different architectures for Bangla handwritten 1–10 digits.

**Figure 12:**
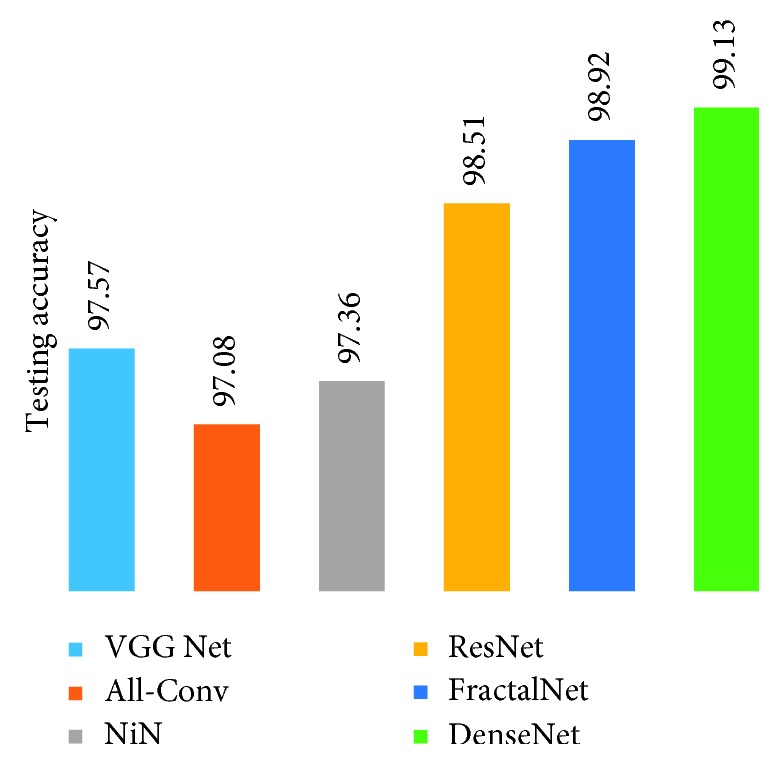
Testing accuracy for Bangla handwritten digit recognition.

Figure 10 shows the training loss of all DCNN models during 250 epochs. It can be observed that FractalNet and DenseNet converge faster compared with other networks, and worst convergence is obtained to be for the All-Conv Network. The validation accuracy is shown in Figure 11, where DenseNet and FractalNet show better recognition accuracy among all DCNN models. Finally, the testing accuracy of all the DCNN models is shown in Figure 12. From the result, it can be clearly seen that DenseNet provides the best recognition accuracy compared with other networks.

### 4.2. Bangla Handwritten Alphabet-50

**Figure 13:**
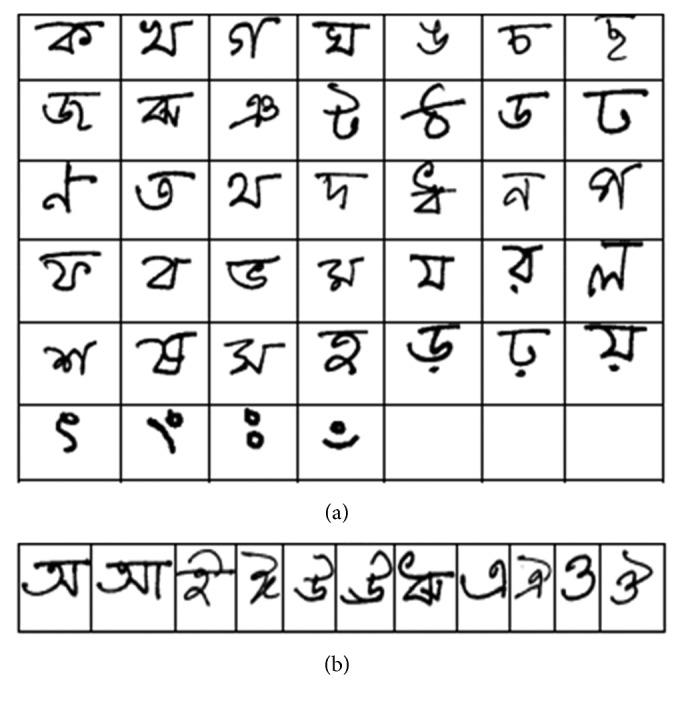
Example images of handwritten characters: (a) Bangla consonant characters and (b) vowels.

**Figure 14:**
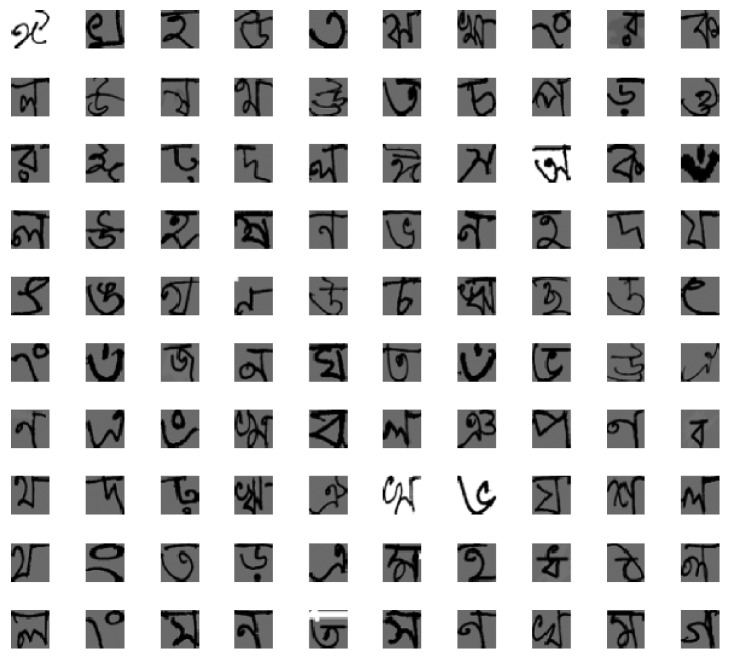
Randomly selected handwritten characters of Bangla alphabets from Bangla handwritten Alphabet-50 dataset.

In our implementation, the basic fifty alphabets including 11 vowels and 39 consonants are considered. The samples of 39-consonant and 11-vowel characters are shown in Figures 13(a) and 13(b), respectively. The Alphabet-50 dataset contains 15,000 samples, where 12,000 are used for training and the remaining 3,000 samples are used for testing. Since the dataset contains samples with different dimensions, we rescale all input images to 32 × 32 pixels for better fitting to the convolutional operation. Some randomly selected samples from this database are shown in Figure 14.

**Figure 15:**
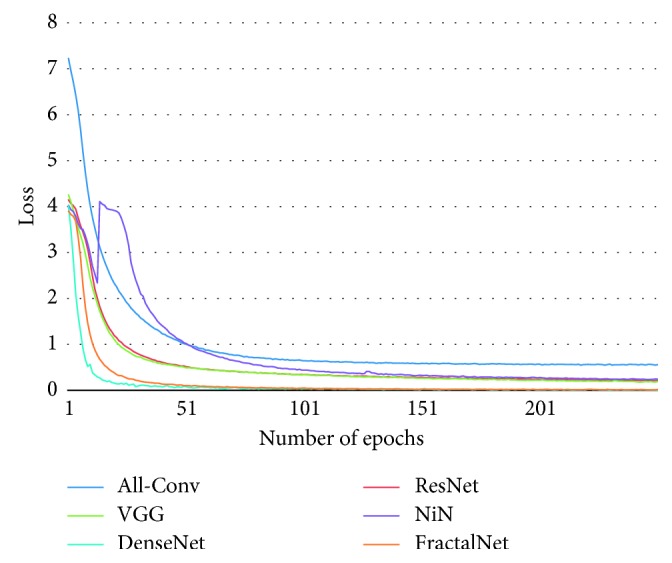
Training loss of different DCNN models for Bangla handwritten Alphabet-50.

**Figure 16:**
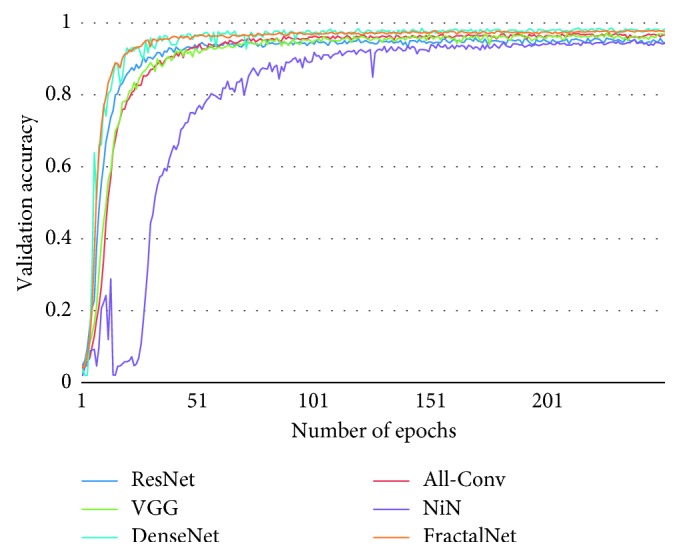
The validation accuracy of different architectures for Bangla handwritten Alphabet-50.

The training loss for different DCNN models is shown in Figure 15. It is clear that the DenseNet shows the best convergence compared with the other DCNN approaches. Similar to the previous experiment, All-Conv shows the worst convergence behavior. In addition, an unexpected convergence behavior is observed in the case of NiN model. However, all DCNN models tend to converge after 200 epochs. The corresponding validation accuracy on Alphabet-50 is shown in Figure 16. DenseNet again shows superior validation accuracy compared with other DCNN approaches.

**Figure 17:**
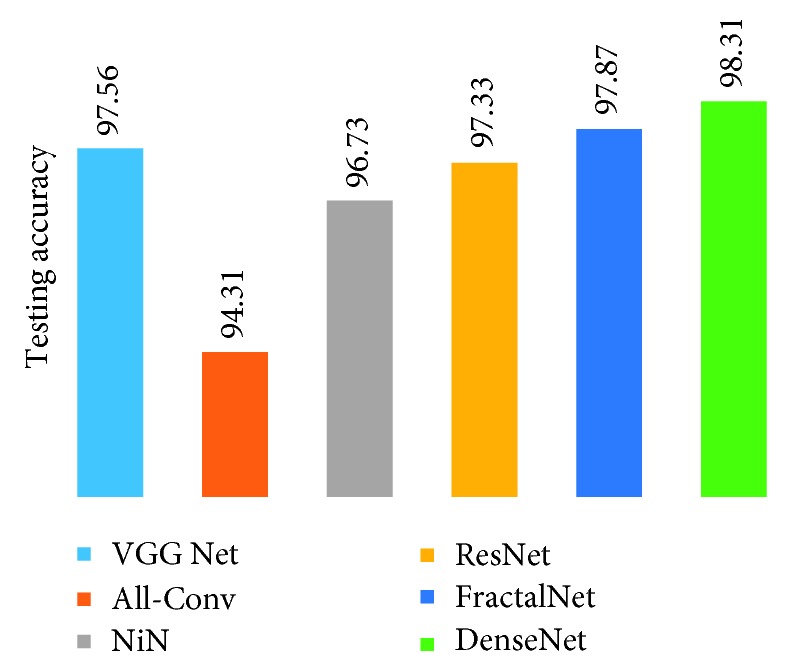
Testing accuracy for handwritten 50-alphabet recognition using different DCNN techniques.

Figure 17 shows the testing results on handwritten Alphabet-50. The DenseNet shows the best testing accuracy with a recognition rate of 98.31%. On the other hand, the All-Conv Net provides around 94.31% testing accuracy, which is the lowest testing accuracy among all the DCNN models.

### 4.3. Bangla Handwritten Special Characters

**Figure 18:**
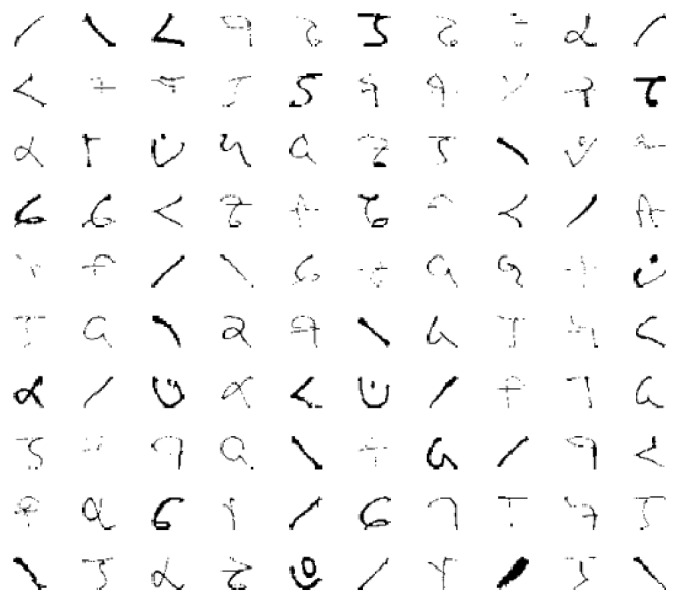
Randomly selected images of special character from the dataset.

There are several special characters (SpecialChar-13) which are equivalent to representations of vowels that are combined with consonants for making meaningful words. In our evaluation, we use 13 special characters which are for 11 vowels and two additional special characters. Some samples of Bangla special characters are shown in Figure 18. It can be seen that the quality of the samples is poor, and significant variation in the same symbols makes this recognition task even difficult.

**Figure 19:**
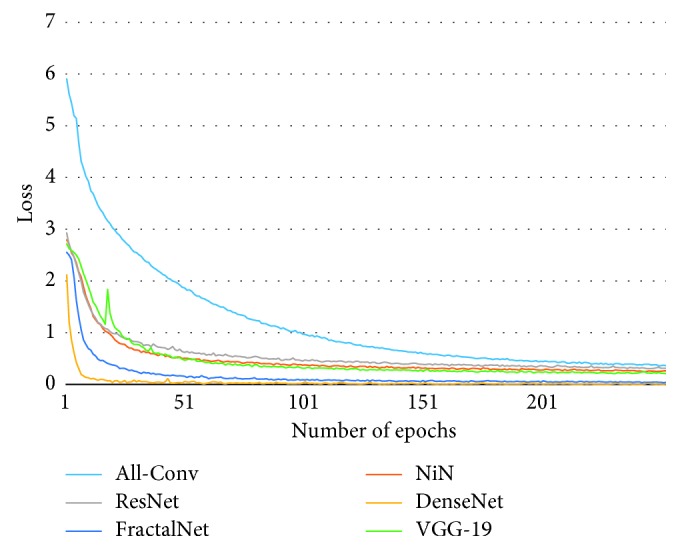
Training loss of different architectures for Bangla 13 special characters (SpecialChar-13).

**Figure 20:**
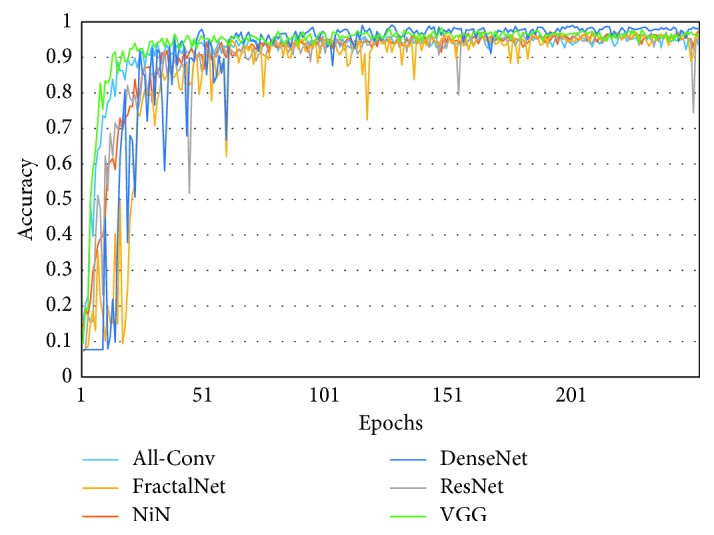
Validation accuracy of different architectures for Bangla 13 special characters (SpecialChar-13).

**Figure 21:**
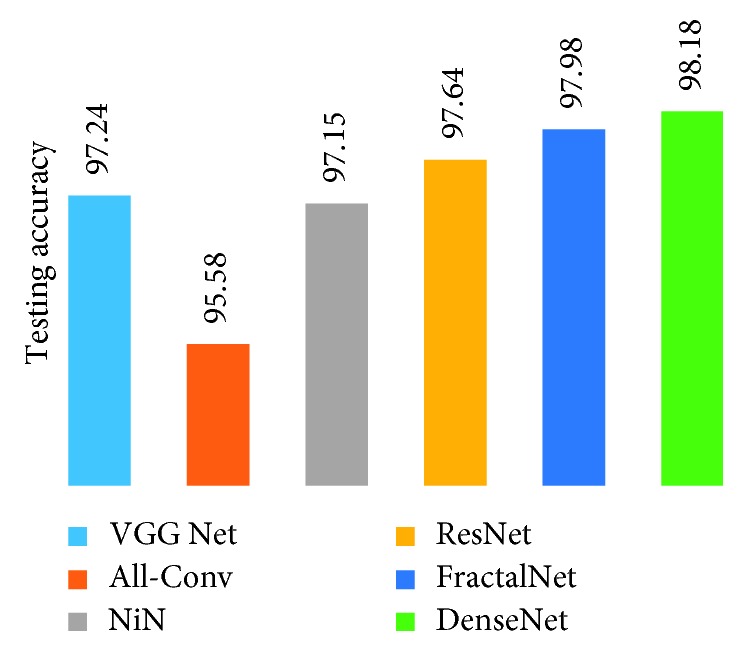
Testing accuracy of different architectures for Bangla 13 special characters (SpecialChar-13).

The training loss and validation accuracy for SpecialChar-13 are shown in Figures 19 and 20, respectively. From these results, it can be seen that DenseNet provides better performance with lower loss and with the highest validation accuracy among all DCNN models.  Figure 21 shows the testing accuracy of DCNN model for SpecialChar-13 dataset. It is observed from Figure 21 that DenseNet shows the highest testing accuracy with lowest training loss and it converges very fast. On the other hand, VGG-19 network shows promising recognition accuracy as well.

### 4.4. Performance Comparison

The testing performance is compared to several existing methods. The results are presented in Table 3. The experimental results show that the modern DCNN models including DenseNet, FractalNet, and ResNet provide better testing accuracy against the other deep learning approaches and the previously proposed classical methods. In general, the DenseNet provides 99.13% testing accuracy for handwritten digit recognition, which is the best accuracy that has been publicly reported to the best our knowledge. In case of a 50-alphabet recognition, DenseNet yields 98.31% recognition accuracy, which is almost 2.5% better than the method in [55]. As far as we know, this is the highest accuracy for handwritten Bangla 50-alphabet recognition. In addition, on 13 special character recognition task, DCNNs show promising recognition accuracy, especially DenseNet achieves the best accuracy which is 98.18%.

### 4.5. Parameter Evaluation

For impartial comparison, we have trained and tested the networks with the optimized same number of parameters as in the references.  Table 4 shows the number of parameters used for different networks for 50-alphabet recognition. The number of network parameters for digits and special character recognition was the same except the number of neurons in the classification layer.

### 4.6. Computation Time

We also calculate computational cost for all methods, although the computation time depends on the complexity of the architecture.  Table 5 presents the computational time per epoch (in second) during training of all the networks for Digit-10, Alphabet-50, and SpecialChar-13 recognition task. From Table 5, it can be seen that DenseNet takes the longest time during training due to its dense structure but yields the best accuracy.

## 5. Conclusions

In this research, we investigated the performance of several popular deep convolutional neural networks (DCNNs) for handwritten Bangla character (e.g., digits, alphabets, and special characters) recognition. The experimental results indicated that DenseNet is the best performer in classifying Bangla digits, alphabets, and special characters. Specifically, we achieved recognition rate of 99.13% for handwritten Bangla digits, 98.31% for handwritten Bangla alphabet, and 98.18% for special character recognition using DenseNet. To the best of knowledge, these are the best recognition results on the CMATERdb dataset. In future, some fusion-based DCNN models, such as Inception Recurrent Convolutional Neural Network (IRCNN) [47], will be explored and developed for handwritten Bangla character recognition.

## Figures and Tables

**Table 1:** Parameter specification in All-Conv model.

Layers	Operations	Feature maps	Size of feature maps	Size of kernels	Number of parameters
Inputs		32 × 32 × 3			
C_1_	Convolution	128	30 × 30	3 × 3	3,456
C_2_	Convolution	128	28 × 28	3 × 3	147,456
S_1_	Max-pooling	128	14 × 14	2 × 2	N/A
C_3_	Convolution	256	12 × 12	3 × 3	294,912
C_4_	Convolution	256	10 × 10	3 × 3	589,824
S_2_	Max-pooling	256	5 × 5	2 × 2	N/A
C_5_	Convolution	512	3 × 3	3 × 3	1,179,648
C_6_	Convolution	512	3 × 3	1 × 1	262,144
GAP_1_	GAP	512	3 × 3	N/A	N/A
Outputs	Softmax	10	1x1	N/A	5,120

**Table 2:** Statistics of the database used in our experiment.

Dataset	# training samples	# testing samples	Total samples	Number of classes
Digit-10	4000	2000	6000	10
Alphabet-50	12,000	3,000	15,000	50
SpecialChar-13	2196	935	2231	13

**Table 3:** The testing accuracy of VGG-16 Network, All-Conv Network, NiN, ResNet, FractalNet, and DenseNet on Digit-10, Alphabet-50, and SpecialChar-13 and comparison against other existing methods.

Types	Methods	Digit-10 (%)	Alphabet-50 (%)	SpecialChar-13 (%)
Existing approaches	MLP [7]	96.67	—	—
	MPCA + QTLR [56]	98.55	—	—
	GA [22]	97.00	—	—
	LeNet + DBN [57]	98.64	—	—
	VGG Net [9]	97.57	97.56	96.15

DCNN	All-Conv [10]	97.08	94.31	95.58
	NiN [11]	97.36	96.73	97.24
	ResNet [12]	98.51	97.33	97.64
	FractalNet [13]	98.92	97.87	97.98
	DenseNet [14]	**99.13**	**98.31**	**98.18**

**Table 4:** Number of parameter comparison.

Models	Number of parameters
VGG-16 [9]	∼8.43M
All-Conv Net [10]	∼2.26M
NiN [11]	∼2.81M
ResNet [12]	∼5.63M
FractalNet [13]	∼7.84M
DenseNet [14]	∼4.25M

**Table 5:** Computational time (in sec) per epoch for different DCNN models on Digit-10, Alphabet-50, and SpecialChar-13.

Models	Digit-10	Alphabet-50	SpecialChar-13
VGG-16 [9]	32	83	15
All-Conv Net [10]	7	23	4
NiN [11]	9	27	5
ResNet [12]	64	154	34
FractalNet [13]	32	102	18
DenseNet [14]	95	210	58

## Data Availability

The data used to support the findings of this study are available at https://code.google.com/archive/p/cmaterdb/.
